# Bloodstream infections in a rural hospital in Sierra Leone: a retrospective database study

**DOI:** 10.1099/jmm.0.002014

**Published:** 2025-05-12

**Authors:** Henning Rottmann, Ioana D. Olaru, Emmanuel Marx Kanu, Tom Theiler, Islam M. Kargbo, Laura Kalkman, Martin P. Grobusch, Frieder Schaumburg

**Affiliations:** 1 Institute of Medical Microbiology, University Münster, Domagkstrasse 10, 48149 Münster, Germany; 2 Masanga Medical Research Unit, Tonkolili District, Sierra Leone; 3 Department of Infectious Diseases, Centre of Tropical Medicine and Travel Medicine, Amsterdam University Medical Centers, Location AMC, University of Amsterdam, Netherlands, Meibergdreef 9, 1105 AZ, Amsterdam; 4 Institute of Tropical Medicine & Deutsches Zentrum für Infektionsforschung, University of Tübingen, Tübingen, Germany; 5 Centre de Recherches Médicales (CERMEL), Lambaréné, Gabon

**Keywords:** Africa, antimicrobial resistance, blood culture, bloodstream infection

## Abstract

**Background.** The health system in Sierra Leone has limited infrastructure to provide data on the epidemiology of infectious diseases and to inform clinical decision-making. The diagnostic and research laboratory capacity at Masanga Teaching Hospital was systematically expanded with microbiology infrastructure-building as one of the centrepieces.

**Objective.** This study aims to report the spectrum of bacterial pathogens from bloodstream infections (BSIs) in a rural hospital in Sierra Leone during the first year after the implementation of a blood culture infrastructure and characterize the detected antimicrobial resistances.

**Patients and methods.** Patients treated at Masanga Hospital (Sierra Leone, March 2023–March 2024) were included in this database analysis if they were tested for BSI (BD BACTEC). Demographic and medical data were recorded for each patient. Antimicrobial susceptibility testing was done following EUCAST clinical guidelines.

**Results.** Of the 340 blood cultures, 34 (10%) were positive for obligate pathogens. The three most frequent pathogens were *Escherichia coli* (*n*=8), followed by *Burkholderia cepacia* complex (*n*=7) and *Salmonella enterica* (*n*=5). Almost all *Klebsiella pneumoniae* (*n*=3/3) and *E. coli* (*n*=7/8) were resistant to third-generation cephalosporins. All four *Staphylococcus aureus* isolates were methicillin susceptible (*mecA* negative). Carbapenem resistance was detected in *Acinetobacter baumannii* complex (*bla*_NDM_)

**Conclusion.** The proportion of positive blood cultures with obligate pathogens (10%) was within the suggested benchmark (5–15%). Gram-negative bacteria dominated the pathogen spectrum of BSI with high resistance rates to third-generation cephalosporins.

## Introduction

Bacterial infections represent a significant cause of global morbidity and mortality, accounting for the second highest cause of death worldwide in 2019, following ischaemic heart disease [1]. Countries of the global South (sub-Saharan Africa and Southeast Asia) shoulder the largest burden [2]. Sepsis has the highest age-standardized incidence rate among bacterial infections in sub-Saharan Africa (1,527.2 per 100,000 population vs. 197.2 per 100,000 population in central Europe) [3]. This is associated with a sepsis-related mortality of 3,492,058 cases in sub-Saharan Africa (Central Europe: 85,807 cases) [3]. Furthermore, the number of deaths associated with antimicrobial resistance (AMR) is also highest in sub-Saharan Africa compared with the rest of the world [4]. Importantly, community-acquired bloodstream infections (BSIs) appear more common in sub-Saharan Africa than hospital-acquired BSI, making BSI a public health problem [5].

Despite these estimates of the burden of sepsis and AMR in Africa, data from many African countries remain poor due to weaknesses in microbiological diagnostics [6,7]. Notably, countries with limited surveillance data and census metrics, such as Sierra Leone, are often underrepresented in region-wide overviews. The health system in Sierra Leone often lacks the basic infrastructure to provide reliable and accurate data on severe bacterial infections including BSI and AMR [7]. Therefore, the diagnostic infrastructure for blood culture testing was established at a regional referral hospital in Sierra Leone. The aim of this study is to understand the bacterial spectrum and AMR of BSI in a hospital in Sierra Leone. The objectives are to report the proportion of bacterial species in BSI, the AMR rates and risk factors for BSI. Although pathogen and AMR statistics on BSI are part of the routine activities of microbiology laboratories in high-income countries, we see an additional clinical value in reporting this information in the context of resource-limited settings.

## Methods

### Study site

Masanga Teaching Hospital (GPS coordinates: 8.750601189945233,–11.83727267976686), a 120-bed government hospital in Tonkolili District (Sierra Leone), treats ~12,000 patients per year. The services include surgery, obstetrics, paediatrics and general healthcare. The medical microbiology laboratory was founded in 2019 and performs cultures (e.g. blood cultures, urine, wound swabs and samples from sterile sites), culture-based identification and antimicrobial susceptibility testing (disc diffusion test) following EUCAST guidelines. In addition, nucleic acid amplification platforms are established for tuberculosis, human immunodeficiency virus (HIV) viral load, Buruli ulcer and sexually transmitted infections. Lateral flow rapid tests are executed for malaria, hepatitis B, hepatitis C, HIV, *Helicobacter pylori* infection and dengue. Microscopy is performed for malaria and sickle cell diagnosis. The microbiology laboratory has an internal quality assurance system, including regular external quality assessments.

### Study population

Patients admitted to Masanga Hospital, Sierra Leone, between March 2023 and March 2024 from whom blood cultures were collected were included consecutively. Test requests for blood cultures were sent by the treating physician, and the decision for testing was based solely on the physician’s judgement. Duplicate samples, follow-up samples (e.g. to test for persistent bacteraemia during treatment as recommended for *Staphylococcus aureus* bacteraemia) or blood culture bottles filled with fluids other than blood (e.g. ascites and pleural effusions) were excluded.

### Microbiological methods

Blood cultures (BD BACTEC™ Plus Aerobic/F-Medium or BD BACTEC™ Plus Peds/F-Medium, BD, Becton Dickinson GmbH, Heidelberg, Germany) were taken by a trained phlebotomy team. Due to limited resources, only one aerobic blood culture bottle per patient was taken with a few exceptions (i.e. follow-up blood cultures in *S. aureus* bacteraemia). Bottles were incubated for 5 days (10 days if HIV was previously detected), at 35±1 °C ambient air in an automatic blood culture system (Bactec FX40, BD).

Species identification and antimicrobial susceptibility testing were done at the study site and confirmed at the Institute of Medical Microbiology, Münster, Germany, using matrix-assisted laser desorption ionization time-of-flight MS (Sirius one, Bruker, Bremen, Germany). Antimicrobial susceptibility testing was confirmed with VITEK II automated system (bioMérieux, Marcy L’Étoile, France) using EUCAST clinical breakpoints.

### Isothermal amplification

Carbapenem resistance in *Enterobacterales* and *Acinetobacter* sp. was confirmed by isothermal amplification of *bla*_KPC_, *bla*_NDM_, *bla*_VIM_, *bla*_OXA-48_ and *bla*_OXA-181_ (eazyplex® SuperBug CRE, AmplexDiagnostics, Gars-Bahnhof, Germany). *Salmonella enterica* was tested for serovars Typhi, Paratyphi and Cholerasuis (eazyplex® TyphiTyper, AmplexDiagnostics).

### Statistical analysis

Descriptive statistics were performed with ‘R’ (version 4.4.1, R Foundation for Statistical Computing, Vienna, Austria). Proportions were compared using the chi-square test or Fisher’s exact test, if appropriate. Antimicrobial agents were categorized based on their chemical structure. Beta-lactams were further subdivided into penicillins, cephalosporins and carbapenems.

## Results

After the exclusion of 16 duplicate samples, a total of 340 blood cultures were included in the final analysis. The median age of the patients was 17 years, with 46% (*n*=155/340) being male and 6% (*n*=21/288) of those tested having an HIV infection (Table 1). Of the 340 patients, 103 (38%) were empirically treated with an antimicrobial agent. The most frequent treatment was ceftriaxone (*n*=68/103), followed by metronidazole (*n*=35/103) and gentamicin (*n*=24/103). Metronidazole and gentamicin were only given in combination. Treatment with a single antimicrobial agent was the exception (*n*=29/103). The most frequently suspected source of infection was skin and soft tissue infections (*n*=45/340), followed by respiratory tract infections (*n*=39/340) and urinary tract infections (*n*=39/340). In 109 patients, the probable source was unknown.

**Table 1.** Demographic characteristics of patients with BSIs in Sierra Leone

	BSI
Included patients (*n*)	340
Children and adolescents (<18 years) [% (*n*)]	51.8% (176)
Adults (≥18 years) [% (*n*)]	48.2% (164)
Median age [years (IQR)]	17 (3–33)
Proportion of females [% (*n*)]	54% (185)
Proportion of people living with HIV [% (*n*)]	7%* (21)

*Only 288 patients in the BSI group had a documented HIV status.

IQR: interquartile range.

Of the 340 blood cultures, 64 (19%) were positive including 10% with an obligate pathogen (*n*=34/340). Likely contaminants were detected in 5% (*n*=16/340) cultures (Table S1, available in the online Supplementary Material). The organism could not be identified/cultured in 14 (4%) of cultures (Table S1). Among the 34 identified pathogens, the most frequently isolated were *Escherichia coli* (*n*=8), followed by *Burkholderia cepacia* complex (*n*=7), *S. enterica* (*n*=5, incl. one *Salmonella* Typhi and four non-typhoidal *Salmonella* isolates), *S. aureus* (*n*=4) and *Klebsiella pneumoniae* (*n*=3). A list of all identified organisms is provided in Table S1.

The majority of *K. pneumoniae* (*n*=3/3) and *E. coli* (*n*=7/8) had resistance to third-generation cephalosporins, leaving piperacillin/tazobactam (*n*=4/11 resistant) and carbapenems (all susceptible) as therapeutic options (Table S2). The majority of *K. pneumoniae* and *E. coli* were resistant to ciprofloxacin (*n*=8/11). All four *S. aureus* isolates were methicillin susceptible. The *Acinetobacter baumannii* complex isolate was meropenem resistant (*bla*_NDM_ positive). The *Salmonella* Typhi isolate was susceptible to ciprofloxacin and ceftriaxone. The cumulative resistance profile of bacteria in Masanga indicates that 19 out of 33 (58%) antimicrobial susceptibility test results demonstrated resistance to three or more antimicrobial agents belonging to different classes (Table 2).

**Table 2.** Number of resistances to antibiotic classes (R) of bacterial isolates from blood cultures in Sierra Leone

Species (*n*)	R0 [% (*n*)]	R1 [% (*n*)]	R2 [% (*n*)]	R3 [% (*n*)]	R4 [% (*n*)]	R≥5 [% (*n*)]
*Escherichia coli* (8)	–	–	13 (1)	–	–	88 (7)
*Klebsiella pneumoniae* (3)	–	–	33 (1)	33 (1)	–	33 (1)
*Enterobacter cloacae* complex (2)	–	–	–	–	50 (1)	50 (1)
*Salmonella enterica* (4)	–	25 (1)	–	50 (2)	25 (1)	–
*Salmonella enterica* Typhi (1)	–	–	100 (1)	–	–	–
*Pseudomonas aeruginosa* (1)	–	–	–	–	–	100 (1)
*Acinetobacter baumannii* complex (1)	–	–	–	100 (1)	–	–
*Burkholderia cepacia* complex (7)	100 (7)	–	–	–	–	–
*Staphylococcus aureus* (4)	–	–	50 (2)	25 (1)	–	25 (1)
*Streptococcus pyogenes* (1)	–	100 (1)	–	–	–	–
*Enterococcus faecium* (1)	–	–	–	100 (1)	–	–
Total (33)	21 (7)	6 (2)	15 (5)	18 (6)	6 (2)	33 (11)

R0–R≥5: number of resistances to antibiotic classes. Beta-lactams are further subdivided into penicillins, cephalosporins and carbapenems.

We tested if certain risk factors are associated with a positive culture. The median age was 17 (interquartile range [IQR]: 10.3–28.5) among those with a positive blood culture with an obligate pathogen and 17 (IQR: 2.5–33.8) among those with negative or contaminated blood cultures (*P*=0.4). A positive blood culture with an obligate pathogen was neither associated with sex (odds ratio [OR]=1.2, 95 % CI: 0.6–2.5, *P*=0.6) nor with HIV infection (OR=1.5, 95 % CI: 0.3–4.7, *P*=0.6). Antimicrobial therapy prior to blood culture collection was not associated with a positive blood culture of an obligate pathogen (OR=0.7, 95 % CI: 0.3–1.7, *P*=0.5).

## Discussion

The proportion of true positive blood cultures (10%) is within the suggested benchmark (5–15% [8]), suggesting that the indication for taking a blood culture is appropriate in this particular setting.

The contamination rate in our setting (5%) is close to the target of ≤3% contaminated blood cultures, although a universal threshold of ≤1% is also now suggested [9]. These numbers should be interpreted with caution as the testing strategy in our setting (1 aerobic bottle per patient) differs from the general recommendation (≥2 pairs of aerobic and anaerobic bottles). There is now evidence that single site sampling (as in our case) has lower contamination rates compared to multiple site sampling to obtain ≥2 pairs of blood culture bottles [10]. Despite the limited sample size, the high prevalence of AMR among *Enterobacterales*, especially to third-generation cephalosporins, is worrisome. This high cephalosporin resistance is in line with one study from Sierra Leone, which demonstrated a high prevalence of extended-spectrum beta-lactamase (ESBL)-producing *Enterobacterales* in postmortem blood samples from stillbirths and children under 5 years of age (*E. coli*: 94.1%, *K. pneumoniae*: 67.5%) [11]. An infection with ESBL-producing *Enterobacterales* requires an escalation of antimicrobial treatment with carbapenems, which are rarely available and affordable in low-resource settings. For some cases of BSI with ESBL producers, piperacillin/tazobactam could still be a treatment option (Table S2), which can be suggested at least for patients with ‘low-risk, non-severe infections’ [12].

The high proportion of BSI with *B. cepacia* complex (Table S1) is uncommon and associated with a cluster of *B. cepacia* complex infections at one ward during the study [13].

This study has limitations. First, the sample size is small and limits the conclusions, especially regarding resistance rates. Nevertheless, the data are considered valuable because they offer initial insights into the epidemiology of BSI in a country with limited infrastructure for culture-based microbiological diagnostic. Second, we did not differentiate viridans streptococci and might underreport cases of endocarditis or dental abscesses, which can have devastating outcomes in our setting [14]. Third, since only one bottle was taken per patient, we are unable to reliably distinguish contamination from true infection.

## Conclusion

The proportion of positive blood cultures with obligate pathogens (10%) was within the suggested benchmark (5–15 %). Gram-negative bacteria dominate the pathogen spectrum of BSI in our setting with high resistance rates to third-generation cephalosporins and the detection of *bla*_NDM_.

## Supplementary material

10.1099/jmm.0.002014 Supplementary Material 1.

## Abbreviations

AMR: antimicrobial resistance
BSI: bloodstream infection
ESBL: extended-spectrum beta-lactamase
