# Supplementary material for: Bloodstream infections in a rural hospital in Sierra Leone: a retrospective database study
Source: J Med Microbiol. 2025 May 12;74(5):002014. doi: 10.1099/jmm.0.002014 (PMC12069813; doi:10.1099/jmm.0.002014)
Supplement: Supplementary Material 1. [file jmm-74-02014-s001.pdf]

Supplement

Title:

Bloodstream infections in a rural hospital in Sierra Leone: A retrospective database study

Table S1: Organisms isolated from blood cultures

**Pathogens**	N=64
Escherichia coli	8
Burkholderia cepacia complex	7
Non-typhoidal *Salmonella enterica*	4
Staphylococcus aureus	4
Klebsiella pneumoniae	3
Enterobacter cloacae complex	2
Acinetobacter baumanii complex	1
Aeromonas caviae	1
Enterococcus faecium	1
Pseudomonas aeruginosa	1
Salmonella enterica Typhi	1
Streptococcus pyogenes	1
**Probable contaminants**	
Coagulase-negative staphylococci	10
Acinetobacter guilouiae	2
Bacillus cereus	1
Pasteurella pneumotropica	1
Microbacterium paraoxydans	1
Pseudomonas mendocina	1
**Unknown**[a]	14

[a]14 isolates could not be re-cultured

Table S2: Antimicrobial susceptibility of obligate pathogen bacteria from bloodstream infections, Sierra Leone

ID	Species	Penicillin	Ampicillin	Amp./sulb.	Piperacillin	Pip./taz.	Cefazolin	Cefoxitin	Cefuroxime	Cefotaxim	Ceftazidim	Ertapenem	Imipenem	Meropenem	Ciprofloxacin	Gentamicin	Tobramycin	Trim./sulfa.	Erythromycin	Clindamycin	Tetracycline	Tigecycline	Glycopeptides	Linezolid	Rifampicin
60008893	*ESCHERICHIA COLI*	NA	R	R	R	R	NA	NA	R	R	R	S	S	S	R	R	NA	R	NA	NA	NA	S	NA	NA	NA
60009462	*ESCHERICHIA COLI*	NA	R	R	R	R	NA	NA	S	S	S	S	S	S	I	S	NA	R	NA	NA	NA	S	NA	NA	NA
60009452	*ESCHERICHIA COLI*	NA	R	R	R	S	NA	NA	R	R	R	S	S	S	R	R	NA	R	NA	NA	NA	S	NA	NA	NA
60009472	*ESCHERICHIA COLI*	NA	R	R	R	S	NA	NA	R	R	R	S	S	S	R	R	NA	R	NA	NA	NA	S	NA	NA	NA
60009475	*ESCHERICHIA COLI*	NA	R	R	R	S	NA	NA	R	R	R	S	S	S	R	R	NA	R	NA	NA	NA	S	NA	NA	NA
60009487	*ESCHERICHIA COLI*	NA	R	R	R	S	NA	NA	R	R	R	S	S	S	R	R	NA	R	NA	NA	NA	S	NA	NA	NA
60009489	*ESCHERICHIA COLI*	NA	R	R	R	S	NA	NA	R	R	R	S	S	S	R	R	NA	R	NA	NA	NA	S	NA	NA	NA
60009490	*ESCHERICHIA COLI*	NA	R	R	R	S	NA	NA	R	R	R	S	S	S	R	R	NA	R	NA	NA	NA	S	NA	NA	NA
60009433	*K. PNEUMONIAE*	NA	R	R	R	R	NA	NA	R	R	R	S	S	S	R	R	NA	R	NA	NA	NA	NA	NA	NA	NA
60009583	*K. PNEUMONIAE*	NA	R	R	R	R	NA	NA	R	R	R	S	S	S	I	R	NA	S	NA	NA	NA	NA	NA	NA	NA
60009450	*K. PNEUMONIAE*	NA	R	R	R	S	NA	NA	R	R	R	S	S	S	I	S	NA	S	NA	NA	NA	NA	NA	NA	NA
60008298	*E. CLOACAE* COMPLEX	NA	NA	R	R	R	NA	NA	NA	R	R	S	S	S	R	R	NA	R	NA	NA	NA	NA	NA	NA	NA
NA	*E. CLOACAE* COMPLEX	NA	NA	NA	NA	NA	NA	NA	NA	R	NA	NA	NA	NA	R	R	NA	R	NA	NA	NA	NA	NA	NA	NA
60008324	*SALMONELLA ENTERICA*	NA	R	R	S	S	NA	NA	NA	S	S	S	S	S	S	R	NA	R	NA	NA	NA	NA	NA	NA	NA
60009464	*SALMONELLA ENTERICA*	NA	S	S	S	S	NA	NA	NA	S	S	S	S	S	S	R	NA	S	NA	NA	NA	NA	NA	NA	NA
60009494	*SALMONELLA ENTERICA*	NA	R	R	R	S	NA	NA	NA	S	S	S	S	S	R	S	NA	R	NA	NA	NA	NA	NA	NA	NA
60009495	*SALMONELLA SER.TYPHI*	NA	S	S	S	S	NA	NA	NA	S	S	S	S	S	S	R	NA	R	NA	NA	NA	NA	NA	NA	NA
60009500	*SALMONELLA ENTERICA*	NA	R	R	R	R	NA	NA	NA	S	S	S	S	S	R	R	NA	R	NA	NA	NA	NA	NA	NA	NA
60008754	*PSEUDOMONAS AERUGINOSA*	NA	NA	NA	R	R	NA	NA	NA	NA	R	NA	I	S	R	NA	R	NA	NA	NA	NA	NA	NA	NA	NA
60009435	*A. BAUMANII* COMPLEX	NA	NA	NA	NA	NA	NA	NA	NA	NA	NA	NA	R	R	I	R	R	R	NA	NA	NA	NA	NA	NA	NA
60008297	*BURKHOLDERIA CENOCEPACIA*	NA	NA	NA	NA	NA	NA	NA	NA	NA	NA	NA	NA	S	NA	NA	NA	S	NA	NA	NA	NA	NA	NA	NA
60008299	*BURKHOLDERIA CEPACIA*	NA	NA	NA	NA	NA	NA	NA	NA	NA	NA	NA	NA	S	NA	NA	NA	S	NA	NA	NA	NA	NA	NA	NA
60008338	*BURKHOLDERIA CEPACIA*	NA	NA	NA	NA	NA	NA	NA	NA	NA	NA	NA	NA	S	NA	NA	NA	S	NA	NA	NA	NA	NA	NA	NA

ID	Species	Penicillin	Ampicillin	Amp./sulb.	Piperacillin	Pip./taz.	Cefazolin	Cefoxitin	Cefuroxime	Cefotaxim	Ceftazidim	Ertapenem	Imipenem	Meropenem	Ciprofloxacin	Gentamicin	Tobramycin	Trim./sulfa.	Erythromycin	Clindamycin	Tetracycline	Tigecycline	Glycopeptides	Linezolid	Rifampicin
60008339	*BURKHOLDERIA CEPACIA*	NA	NA	NA	NA	NA	NA	NA	NA	NA	NA	NA	NA	S	NA	NA	NA	S	NA	NA	NA	NA	NA	NA	NA
60009418	*BURKHOLDERIA CEPACIA*	NA	NA	NA	NA	NA	NA	NA	NA	NA	NA	NA	NA	S	NA	NA	NA	S	NA	NA	NA	NA	NA	NA	NA
60008769	*BURKHOLDERIA CEPACIA*	NA	NA	NA	NA	NA	NA	NA	NA	NA	NA	NA	NA	S	NA	NA	NA	S	NA	NA	NA	NA	NA	NA	NA
60009428	*BURKHOLDERIA CEPACIA*	NA	NA	NA	NA	NA	NA	NA	NA	NA	NA	NA	NA	S	NA	NA	NA	S	NA	NA	NA	NA	NA	NA	NA
60008750	*AEROMONAS CAVIAE*	NA	NA	NA	NA	NA	NA	NA	NA	NA	NA	NA	NA	NA	NA	NA	NA	NA	NA	NA	NA	NA	NA	NA	NA
60008755	*S. AUREUS*	R	NA	S	NA	S	S	S	NA	NA	NA	NA	S	S	NA	S	NA	R	R	R	S	S	S	S	S
60009434	*S. AUREUS*	R	NA	S	NA	S	S	S	NA	NA	NA	NA	S	S	NA	S	NA	R	S	S	S	S	S	S	S
NA	*S. AUREUS*	R	NA	S	NA	S	S	S	NA	NA	NA	NA	S	S	NA	NA	NA	S	S	R	R	NA	NA	NA	NA
NA	*S. AUREUS*	R	NA	S	NA	S	S	S	NA	NA	NA	NA	S	S	NA	NA	NA	S	S	S	R	NA	NA	NA	NA
60009451	*S. PYOGENES*	S	NA	S	S	NA	S	NA	NA	NA	NA	S	S	S	NA	NA	NA	S	R	S	S	S	S	S	S
60008311	*E. FAECIUM*	NA	R	R	R	R	NA	NA	NA	NA	NA	NA	R	NA	R	S	NA	NA	NA	NA	NA	S	S	S	NA
